# IMPLICIT AND EXPLICIT SPIRITUALITY IN THE LIVES OF TRANSGENDER AND GENDER-EXPANSIVE OLDER ADULTS

**DOI:** 10.1093/geroni/igad104.1551

**Published:** 2023-12-21

**Authors:** Vanessa Fabbre, Courtney Taylor, Stef Sloan, Eleni Gaveras

**Affiliations:** Washington University in St. Louis, St. Louis, Missouri, United States; Brown School of Social Work, Washington University in St. Louis, St. Louis, Missouri, United States; University of Kansas, Lawrence, Kansas, United States; Washington University in St Louis, St. Louis, Missouri, United States

## Abstract

Religion and spirituality for transgender and gender expansive people (whom we refer to collectively as trans) are complicated by mainstream religions’ history of stigmatizing and marginalizing sexual and gender minorities. We conducted an interpretive content analysis of biographical interviews with 88 trans older adults from across the United States, applying six tenets of spiritual psychotherapy to their life narratives. Our findings suggest that some trans older adults’ spirituality is experienced both implicitly and explicitly. Implicit spirituality reflects the ways in which meaning, purpose, and connection in one’s life are nurtured with respect to one’s gender identity. Explicit spirituality reflects the process of consciously renegotiating one’s spiritual beliefs and religious practices to validate one’s gender identity and place in society. This knowledge is potentially helpful for gerontological practitioners who seek to nurture trans people’s spirituality and well-being as they age.

